# Sterilization process control in health centre Bijeljina

**DOI:** 10.1186/2047-2994-4-S1-P48

**Published:** 2015-06-16

**Authors:** R Jelisic, Z Maksimovic, S Mijatovic, SS Peric, C Radovanovic

**Affiliations:** 1 Health Centre Bijeljina, Bijeljina, Bosnia and Herzegovina

## Introduction

Within health care systems of less developed countries, such as Rep. of Srpska, Bosnia and Herzegovina, there is a lack of directives and guidelines within the area of infectious disease control. This especially applies to primary level institutions which are therefore required to develop and conduct measures of prevention and elimination of intrahospital infections independently.

## Objectives

Our goal is to show the importance of establishing general sterilization monitoring procedures in primary health care institutions of less developed countries that lack the regulatory guidelines within this field.

## Methods

The method and process of sterilization have been defined by the guidelines given in the Sterilization process, which are mandatory to all services within the institution. Steam sterilization control is conducted through four control categories: Process, Pack, Load and Equipment. Dry heat sterilization control is conducted through two control categories: process and load.

## Results

Health Centre Bijeljina provides primary health care with eleven medical services, operating in the main facility and the units located in rural areas. Within the Centre 30 dry heat sterilizers and 2 autoclaves are being used. We have shown results of biological control of sterilization for period 4 years while the results of process, pack and equipment control were NAD. Load control – 842 spore-testing of dry heat sterilizers were performed and the number of positive test results was 10.57%.

The cause of positive test results in 83.3% cases was the human factor, improper use of equipment and the failure to follow the complete sterilization procedure.

Load control – 182 spore-testing of steam sterilization was conducted and the number of positive test results was 0.

## Conclusion

Based on the test results analysis we came to a conclusion that implementing central sterilization in primary level institutions, with the use of autoclaves, is necessary to enable more efficient and effective sterilization process. The implementation of central sterilization also allows more reliable sterilization monitoring as well as considerable resource savings.

## Disclosure of interest

None declared.

